# Developmental and Behavioral Pediatrics: an emerging subspecialty. Experience of the Instituto da Criança e do Adolescente — ICr-HCFMUSP

**DOI:** 10.1016/j.clinsp.2022.100126

**Published:** 2022-10-23

**Authors:** Ana Paula Scoleze Ferrer, Sandra Josefina Ferraz Ellero Grisi

**Affiliations:** Departamento de Pediatria, Faculdade de Medicina, Universidade de São Paulo, São Paulo, SP, Brazil

**Keywords:** DBP, Developmental and Behavioural Pediatrics

The World Health Organization emphasizes the importance of ensuring full child development to secure the social and economic future of individuals and society.^1^ It is estimated that one in six American children and adolescents have developmental and/or behavioral problems.^2^ In Brazil, even though the estimates are not precise, the increase in suspicion of children with developmental problems and/or delays has increased the interest in this topic in several medical specialties (pediatrics, neurology, psychiatry, phoniatrics), other health disciplines (psychology, speech therapy, occupational therapy, physiotherapy), and other sciences (pedagogy, anthropology, sociology, economics).

In Pediatrics, this field began to expand mainly in the 1960s, with the creation of a section focused on child development and behavior within the American Academy of Pediatrics and training programs affiliated with university centers. However, it was only in 1999 that Developmental and Behavioral Pediatrics (DBP) was recognized as a pediatric subspecialty, later certified by the American Board of Pediatrics in 2002.^3^^,^^4^ DBP is a subspecialty recognized in several countries, but in Brazil, although it is not yet a recognized area of practice, it has been emerging in several centers linked to care, research and teaching. Since 2010, it has been integrated as a scientific department of the Brazilian Society of Pediatrics.^5^

Despite the growing interest, there are still discussions about the definition of its field of action once different specialties work in this area.^6^ That is why the authors have witnessed the creation of services, mainly linked to research, teaching and assistance which bring together the several disciplines involved in child development and behavior and aim to develop the practice and protocols for multidisciplinary attention. The authors have also seen an increase in complaints related to behavior and learning by parents and schools, an increase in suspicions of developmental problems, particularly autism spectrum disorder, and a significant increase in referrals to special therapies, often in the form of judicial reviews, which have impacted public and private healthcare services. Thus, it is important to discuss possible care settings and the organization of care in this field.

This report presents the experience of the Development and Behavior Outpatient Clinic of the *Instituto da Criança e do Adolescente da Faculdade de Medicina da Universidade de São Paulo* (APDC ICr-HCFMUSP) aiming to contribute to the definition and delimitation of the scope of care of the DBP and contribute to the discussion about a model of care.

One of the great challenges in relation to facing complaints of developmental and behavioral problems is to optimize the diagnostic process so that it is not delayed, since early interventions have better results, and accuracy, which avoids the stigmatization of the child as well as unnecessary tests and interventions.

The APDC ICr-HCFMUSP was created as an interdisciplinary in-service for the teaching and training of DBP of the Department of Pediatrics of the FMUSP in 2017, attending to children referred from the HCFMUSP complex or from external requests. The main focus of the APDC is to perform the diagnostic evaluation for the accurate and timely referral to therapies that each child really needs or to perform tests when necessary. In addition, it guides strategies to stimulate child development and parenting and supports families in the therapeutic pathway. For the diagnostic process, the care is multidisciplinary and involves the joint evaluation by a pediatrician trained in this area, a psychologist, a speech therapist, and a psychomotor therapist, in addition to a group of psychopedagogues, when necessary.

The evaluation requires 3–5 appointments and includes extended anamnesis, physical examination, playful interaction with the child, and careful observation of the relationships between child-caregiver (and vice-versa) and child-professional, and their reactions to the environment and forms of play. When necessary, specific assessment instruments and scales are applied. Therefore, the diagnostic process goes beyond the recognition of signs and symptoms and the verification of developmental milestones. It characterizes relational aspects and parenting style and identifies psycho and/or social aggravating factors, following the guiding axes proposed in the “DC 0‒5: Diagnostic Classification Manual for Mental Health Disorders and Childhood Development”.^7^

Since its implementation, approximately 700 appointments have been performed, with growing demand. Patient ages ranged from 13 months to 17 years (mean = 76.4 months), 65% were male. The referrals came from pediatricians (44.2%), other health professionals (25.5%), family (19.1%), or schools (11.2%). The main reasons for referral were: suspected autistic spectrum disorder (29.2%), learning difficulties (16.9%), behavior problems (15.4%), language delays (13.8%), suspected attention deficit hyperactivity disorder (10.8%), global development delays (12.3%), and mood disorders (1.5%). After evaluation, about 35% of the referral reasons were not confirmed (by the change in the initial diagnosis or by discarding the suspicion of a developmental problem). Many of these patients required only guidance on stimuli and parenting. The others were referred to the most appropriate therapies for each case.

Although developmental surveillance is one of the pillars of pediatric practice, the great demand and the greater complexity of the clinical presentation of patients have made it difficult for general pediatricians to act. Then, the DBP represents an area of differentiated performance in the deepening of diagnosis and guidance as well as more precise referrals for more complex cases, supporting decision-making.^8^ This approach contributes to patient recovery and is more cost-effective for the service.

In relation to other specialties, particularly child psychiatry and neuropediatrics, DBP has differentiated training closer to the practice of pediatrics and without the bias of observation that specialization in those areas imposes on the professional. The training as a pediatrician and the greater familiarity with typical development allow the DBP to take a comprehensive approach, including environmental aspects, family relationships, and psychological and socio-cultural aspects. DBP can perform the diagnostic process, guide the promotion of development and parenting, establish a link with community services, and refer to therapies or other specialists in those cases that require them.^6^^,^^8^

Therefore, what differentiates the performance of DBP from a general pediatrician is the depth of the evaluation, while with the other specialties; it would be the scope of action.

## Conflicts of interest

The authors declare no conflicts of interest.
